# Phenolic Profiling and Antioxidant Capacity of *Eugenia uniflora* L. (Pitanga) Samples Collected in Different Uruguayan Locations

**DOI:** 10.3390/foods7050067

**Published:** 2018-04-24

**Authors:** Ignacio Migues, Nieves Baenas, Amadeo Gironés-Vilaplana, María Verónica Cesio, Horacio Heinzen, Diego A. Moreno

**Affiliations:** 1 Facultad de Química, UdelaR, Av. Gral. Flores 2124, 11800 Montevideo, Uruguay; imigues@fq.edu.uy (I.M.); cs@fq.edu.uy (M.V.C.); heinzen@fq.edu.uy (H.H.); 2 CEBAS-CSIC, Food Science and Technology Department, Phytochemistry and Healthy Foods Laboratory, Campus Universitario Espinardo-25, E-30100 Espinardo, Murcia, Spain; nbaenas@cebas.csic.es (N.B.); amadeo.girones@elpozo.com (A.G.-V.)

**Keywords:** *Eugenia uniflora* L., nutraceuticals, antioxidant activity, polyphenols

## Abstract

The use of nutrient-rich foods to enhance the wellness, health and lifestyle habits of consumers is globally encouraged. Native fruits are of great interest as they are grown and consumed locally and take part of the ethnobotanic knowledge of the population. Pitanga is an example of a native fruit from Uruguay, consumed as a jelly or an alcoholic beverage. Pitanga has a red-violet pigmentation, which is a common trait for foods that are a good source of antioxidants. Hence, fruits from different Uruguayan regions were analyzed via miniaturized sample preparation method, HPLC-DAD-ESI/MS^n^ and RP-HPLC-DAD techniques to identify and quantify phenolic compounds, respectively. The antioxidant capacity was evaluated via DPPH and ORAC (Oxygen Radical Absorbance Capacity) assays. A multivariate linear regression was applied to correlate the observed antioxidant capacity with the phenolic content. Furthermore, Principal Components Analysis was performed to highlight characteristics between the various samples studied. The main results indicated differences between northern and southern Uruguayan samples. Delphinidin-3-hexoside was present in southern samples (mean of 293.16 µmol/100 g dry weight (DW)) and absent in the sample collected in the north (sample 3). All the samples contain high levels of cyanidin-3-hexoside, but a noticeable difference was found between the northern sample (150.45 µmol/100 g DW) and the southern sample (1121.98 µmol/100 g DW). The antioxidant capacity (mean ORAC of 56370 µmol Trolox^®^/100 g DW) were high in all the samples compared to the Food and Drug Administration (FDA) database of similar berry-fruits. The results of this study highlight the nutraceutical value of a native fruit that has not been exploited until now.

## 1. Introduction

*Eugenia uniflora* L. belongs to Myrtaceae family and grows naturally in subtropical Latin-American zones [1]. Its cultivation has extended to other regions outside the American continent with similar climatic conditions. It grows mainly in Brazil, which is why it is known as “Brazilian Cherry”, but it is also grown in Argentina, Paraguay and Uruguay [2]. Its fruits have a high carbohydrates content (around 38%) where maltose, lactose and fructose are the main identified compounds [3,4]. It has a high content of vitamin C, vitamin A, riboflavin (B12) and niacin (B3) [4]. Immature fruits show a high content of polyphenols that decreases with maturation [5]. On the other hand, carotenoid content increases with maturation evidenced by an increase in a reddish-orange coloration [6].

Pitanga leaves are often used in Brazilian traditional medicine due to its diuretic, antirheumatic, antifebrile, anti-inflammatory and hypocholesterolemic properties [7,8,9,10]. However, there is little information regarding the medicinal use of the fruit. The fruit has an acidic and sweet flavor and can be consumed fresh, in compotes, jams or juices [6]. Purple fleshed Pitanga fruit in its latest maturation stage has an edible portion (pulp and skin) of 61.76%, a vitamin C content of 38.35 mg/100 g and total anthocyanin content of 29.60 mg/100 g [11]. Additionally, a high content of total phenols (799.80 mg of gallic acid/100 g) and total carotenoids (5.86 μg of β-carotene/g) [4]. Recently, Pitanga juice showed an anti-inflammatory effect on oral gum epithelial cells; these results could be associated to the presence of cyanidin-3-glucoside and oxidoselina-1,3,7(11)-trien-8-one [12,13]. Hence, Pitanga’s beneficial properties could be exploited in the nutraceutical industry.

According to DeFelice a nutraceutical can be defined as “a food or part of it, that has some health benefit, including the prevention and/or treatment of a disease” [14]. Therefore, research has driven evaluation/re-evaluation of foods and their beneficial properties. Antioxidant rich foods have received a lot of interest; promoting their consumption to decrease oxidative stress caused by stress, lack of sleep, poor diet, metabolic problems, etc.

Currently, a large number of different approaches to determine antioxidant activity have been established [15,16,17]. All tests differ in substrates, probes, reaction conditions, instrumentation, and quantification methods. Hence, it is difficult to compare the results obtained by one method or another. Based on the reactions involved, the tests can be further classified into two groups: HAT (Hydrogen Atom Transfer) and SET (Single Electron Transfer) [18]. In the HAT group, antioxidants must donate a hydrogen atom to stabilize the generated free radicals (a synthetic free radical generator and an oxidizable molecular probe are used to evaluate the kinetics of the reaction). SET-type assays involve a redox reaction where the antioxidant must donate an electron to the generated free radical. In both methods the “competition” with the oxidant radical is measured instead of the antioxidant capacity [18].

The ORAC (Oxygen Radical Absorbance Capacity) assay measures the overall antioxidant activity or capacity of a sample’s ability to “quench or neutralize” peroxyl radicals. The peroxyl radicals are reactive species comparable to those ROS (Reactive Oxygen Species) biologically generated in the organism. In the ORAC assay, the peroxyl radicals, generated from the azo-compound AAPH (2,2′-azobis-(2-methylpropionamidine)dihydrochloride) react with fluorescein as a substrate. The fluorescence of the latter compound decreases over time, forming an area under the curve (fluorescence vs. time). In the presence of antioxidant compounds, the area under the curve increases linearly and proportionally to the concentration of antioxidants.

The ORAC assay quantifies via the HAT mechanism and measures the antioxidant activity of polyphenol and non-polyphenolic compounds present in each sample. It is important to note that the antioxidant activity does not have a direct correlation to the polyphenol nature of a sample [19]. The ORAC test reflects the overall capacity or antioxidant activity of a sample, due to the individual components and their additive, synergistic interactions. The ORAC value is usually expressed as micromoles of Trolox^®^ equivalents/100 g of sample. Trolox^®^ is an analogue of vitamin E and is often used as a comparative standard due to its solubility in water [18,20].

The DPPH method name stems from the reactant used (2,2-diphenyl-1-picrylhidracyl) to evaluate the ability of a sample’s antioxidants to “quench or neutralize” a free radical. The DPPH assay utilizes molecules that differ completely from any free radical or reactive species generated by our organism as a source of free radicals. Although the reactant is easy to use, it places this method as a distant analytical approximation of the high reactivity that typically characterizes the ROS generated in biological systems [21]. This method is classified in the SET group. However, can be classified as a HAT-type assay according to the antioxidant present in the sample [13]. In this test the sample is incubated for 35 min and the decay of the absorbance is measured at 515 nm.

Based on previous reports of bioactivity [22] of South-Brazilian Pitanga fruit, the objective of the present work was to extract, identify and quantify the phenolic compounds in mature fruits of *Eugenia uniflora* L. The antioxidant capacity was evaluated via ORAC and DPPH; and the phenolic profiles of *E. uniflora* from different Uruguayan locations were compared. Pitanga is considered a South American native fruit and its consumption is not among the most common in the region. However, its ethnobotanical characteristics, traditional uses and its antioxidant properties highlights its potential to improve the quality of life of those who consume it [23]. Hence, this study seeks to explore and further promote the value of the Uruguayan native Pitanga fruit that has not been exploited until now.

## 2. Materials and Methods

### 2.1. Chemicals

2,2-Diphenyl-1-picrylhidracyl (DPPH), fluorescein, 2,2′-azobis-(2-methylpropionamidine)dihydrochloride (AAPH), monobasic sodium phosphate and dibasic sodium phosphate were obtained from Sigma-Aldrich (Steinheim, Germany). 6-hydroxy-2,5,7,8-tetramethylchroman-2-carboxilic acid (Trolox^®^) was purchased from Fluka Chemika (Neu-Ulm, Germany). Cyanidin-3-*O*-glucoside and rutin were obtained from Polyphenols Laboratories AS (Sandnes, Norway). Ultrapure water was produced using a Millipore Milli-Q^®^ Ultrapure Water Solutions Type 1. All solvents used were HPLC grade from Sigma-Aldrich (Steinheim, Germany).

### 2.2. Samples

The fruits (purple fleshed Pitanga breeding lines) were collected in different Uruguayan locations (described below), they were identified and kept at the Jose Arechavaleta Herbarium in the Faculty of Chemistry, UdelaR, Uruguay (Voucher number MVFQ 4427). Samples 1, 5, 6 & 7 were collected in the north of Montevideo Department (−34.804951, −56.230206) in December 2014, November 2015, December 2015 and April 2016, respectively. Sample 2 was collected in the south of Montevideo Department (−34.884536, −56.073039), sample 3 in Paysandú Department (−32.322604, −58.088243) and sample 4 in Ciudad de la Costa, Canelones Department (−34.799105, −55.908381); samples 2, 3 & 4 were collected in December 2014. All samples were freeze-dried, and the water content calculated by weight before and after lyophilization.

### 2.3. Extraction

Each lyophilized grinded sample (100 mg) was mixed with 1 mL of methanol/water/formic acid (70:29:1, *v*/*v*/*v* ) in a 2 mL conical polypropylene tube (Eppendorf, Madrid, Spain). Then, the samples were vortexed and subjected to indirect sonication in an ultrasound cleaning bath for 60 min (BRANSONIC^®^ Ultrasonic cleaner mod. 5510E-MTH, Ultrasonic frequency: 135 W or 42 KHz ± 6%). The samples were kept overnight at 4 °C and sonicated again for 60 min. The supernatant was separated from the solid residue after centrifugation (9500× *g*, 15 min), filtered using a 0.45 µm PVDF filter (Millex HV13, Millipore, Bedford, MA, USA) and stored at 4 °C until they were analyzed.

### 2.4. Identification of Phenolic Compounds Via HPLC-DAD-ESI/MS^n^ and Quantification Via RP-HPLC-DAD

The identification analyses were carried out using an Agilent HPLC 1100 series model equipped with a photodiode array detector and a mass detector in series (Agilent Technologies, Waldbronn, Germany). It consisted of a binary pump (model G1312A), a degasser (model G1322A), an autosampler (model G1313A), and a photodiode array detector (model G1315B). The HPLC system was controlled by ChemStation for LC 3D Systems software Rev. B.01.03-SR2 (204) (Agilent Technologies Spain S.L., Madrid, Spain). The mass detector was an ion trap spectrometer (model G2445A) equipped with an electrospray ionization interface and was controlled by LC/MS software (Esquire Control Ver. 6.1. Build No. 534.1., Bruker Daltoniks GmbH, Bremen, Germany). The ionization conditions were 350 °C capillary temperature and 4 kV voltage, the nebulizer pressure was 65.0 psi and the nitrogen flow rate was 11 L/min. Full-Scan mass covered the range of *m*/*z* from 100 to 1200. Collision-induced fragmentation experiments were performed in the ion trap using helium as the collision gas, with voltage ramping cycles from 0.3 to 2 V. The mass spectrometry data were acquired in the positive ionization mode for anthocyanins and in the negative ionization mode for other flavonoids. The MS^n^ was carried out in the automatic mode on the more abundant fragment ion in MS^(n-1)^. A Luna C_18_ column (250 × 4.6 mm, 5 µm particle diameter; Phenomenex, Macclesfield, UK) was used. Mobile phase A: water/formic acid (99:1, *v*/*v*), mobile phase B: acetonitrile, initial conditions: 8% solvent B, reaching 15% solvent B at 25 min, 22% at 55 min, and 40% at 60 min, which was maintained isocratic until 70 min. The flow rate was 0.8 mL/min during the whole run, all gradients were linear, and the injection volume was 7 µL. Chromatograms were recorded at 280, 320, 360 and 520 nm.

For quantification experiments the same conditions were applied, except for the injection volume was set to 20 µL and the flow rate was 0.9 mL/min. Anthocyanins were quantified as cyanidin 3-*O*-glucoside at 520 nm and flavonols as rutin at 360 nm.

### 2.5. DPPH Antioxidant Activity Measurements

Experiments were carried out using 96-well micro plates (Nunc, Roskilde, Denmark) and an Infinite^®^ M200 micro plate reader (Tecan, Grödig, Austria). The antioxidant activity was evaluated measuring the change of the absorbance at 515 nm after 35 min of reaction with the radical DPPH˙ (2 µL of the sample + 250 µL of DPPH˙ solution). The results were expressed as µmol of Trolox^®^/100 g dry weight.

### 2.6. ORAC Antioxidant Activity Measurements

The antioxidant assay was performed using black-walled 96-well plates (Nunc, Roskilde, Denmark) and an Infinite^®^ M200 micro plate reader (Tecan, Grödig, Austria). Each well with a final volume of 200 μL. 10 mM phosphate buffer (pH 7.4) was used to prepare 1 μM fluorescein and 250 mM AAPH solutions. Each well received 150 μL of fluorescein solution and 25 μL of phosphate buffer, Trolox^®^ solutions or sample solution to measure the blank, the curve or the samples respectively. The plate was placed into the microplate reader and after 30 min of incubation at 37 °C, 25 μL AAPH solution were added to each well and fluorescence was recorded every 5 min for 120 min using an excitation wavelength of 485 nm and an emission wavelength of 520 nm. ORAC values were calculated using the difference in Areas Under the Fluorescein Decay Curve (AUC) between the blank and a sample. The results were expressed as µmol of Trolox^®^/100 g dry weight.

### 2.7. Statistical Analysis

Data shown are mean values (*n* = 3), subjected to Analysis of Variance (ANOVA) and multiple range test (Tukey’s test), using RStudio software (Version 1.1.383, RStudio, Boston, MA, USA) and InfoStat (Version 2017, Universidad Nacional de Córdoba, Córdoba, Argentina) [24].

## 3. Results and Discussion

### 3.1. Water Content

The results of the water content of the samples are shown in Table 1.

The stress generated by the lack of precipitations, is associated to the increased concentration of sugar and phenolic compounds in grapes [25]. For grapes this enhances fermentation and wine quality. However, in a study where water availability and photosynthetic efficiency of Pitanga plants was evaluated the main influencing factor that determined the quality of the fruits was not water availability, but light exposure [26].

During 2014, Uruguay suffered from excess precipitations, especially in the south of the country (60% above the regular precipitation rate) [27]. However, sample 4 showed a smaller water %, which could be due to its location near the beach, where water availability was less due to the sandy ground. During 2015, Uruguay suffered from slight droughts and higher temperatures at the end of the year, which could be the cause of the decreased water content in sample 6. The first two months of 2016 experienced a lot of rain and consequently, the water content of sample 7 was much higher in comparison to the other studied samples.

### 3.2. Identification and Quantification of Phenolic Compounds

As expected, all samples resulted in similar phenolic profiles. Hence, the different climate situations had no influence, see Table 2. Two anthocyanins were identified, delphinidin-3-hexoside and cyanidin-3-hexoside, the latter was the main anthyocyanin present in all samples. The results are in agreement with previously obtained values by Einbond in 2004 [28]. Thirteen flavonols were identified and myricetin-rhamnoside was the most abundant in all samples. Samples collected from northern Montevideo showed variable levels of total flavonol content (TFC) that ranged from 120 to 860 µmol/100 g dry weight, but the Paysandú sample showed a much higher level of TFC (1050 µmol/100 g dry weight), see Table 3.

The total anthocyanin content (TAC), total quercetin derivatives content (TQC), total myricetin derivatives content (TMC), total flavonol content (TFC), total catechol derivatives content (TCC), total pyrogallol derivatives content (TPC) and total polyphenol content (TPPC) were calculated for each sample and the results are shown in Table 3.

### 3.3. Antioxidant Activity

The results of the antioxidant assays (ORAC and DPPH) are shown in Table 4, the results for both assays are expressed as (µmol Trolox^®^/100 g dry weight).

In general, samples collected in the north of Montevideo showed greater level of ORAC scavenging activity, but similar levels of DPPH activity compared to the other samples. The direct correlation between ORAC-TAC, ORAC-TFC, DPPH-TAC, and DPPH-TFC was not statistically significant (*R*^2^ < 0.3 in all cases). However, the ORAC and DPPH activity of all Pitanga samples were higher compared to other Latin-American berries [29].

All the samples collected in southern Uruguay contained delphinidin together with cyanidin. This is a clear difference with the findings of Celli [5] that described cyanidin as the only occurring anthocyanin in Brazilian Pitanga samples. Northern Uruguay is naturally connected with southern Brazil but the continuity towards southern Uruguay is broken by the Negro river, these results could point out a region related chemo-diversity. Furthermore, three other myricetin derivatives were not identified in the Brazilian samples.

**Figure 1:**
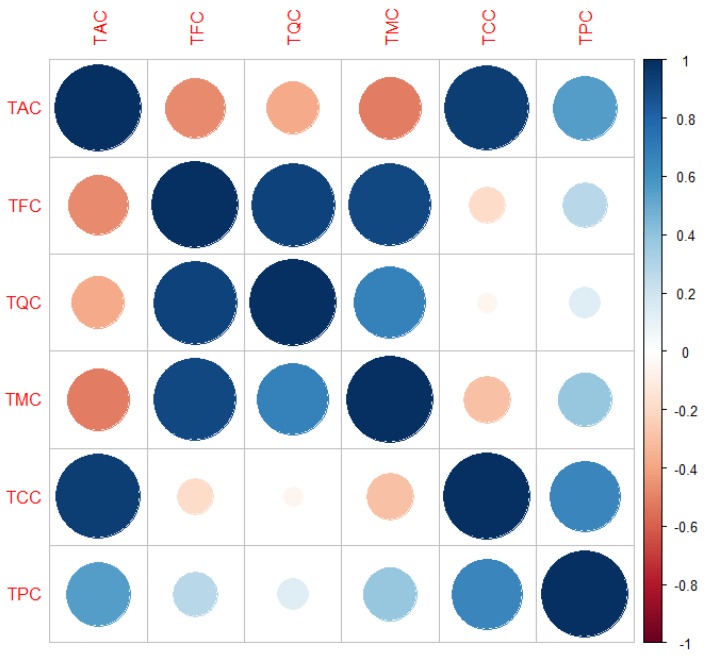
Correlation boxplot between identified and quantified compounds.

Studying the linear correlation (Figure 1) between the identified compounds grouped as it is shown in Table 3, we can see that when the concentration of anthocyanins increased, the flavonol content decreased, that is a negative correlation (shown in red spots, bigger spots represents a stronger correlation). A strong positive correlation between quercetin and myricetin derivatives, and a negative correlation of both flavonol groups to the anthocyanins concentration is shown. When the biosynthesis of anthocyanins is favored, there is less biosynthesis of flavonols as expected from their common biosynthetic pathway, similarly described for bilberries [30].

**Figure 2:**
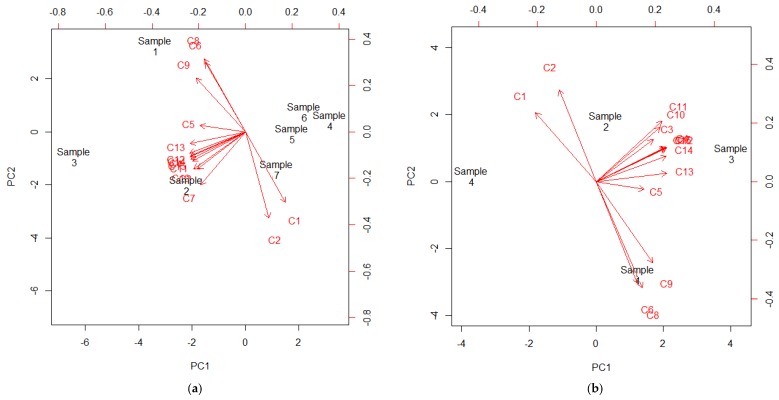
Phenolic content PCA of: (**a**) all samples, and (**b**) samples collected in December 2014.

A PCA was performed using the phenolic content to identify the compounds that weighted the most when classifying the samples. Figure 2a shows that all the samples are grouped together according mainly to PC1 (69.62% of the variance), except for the sample collected in Paysandú (sample 3). Sample 7 is located closer to the anthocyanins than the rest of the samples, mainly because of PC2 (15.68% of the variance).

When we analyze the samples collected in 2014, sample 4 (Canelones) and 2 (south of Montevideo) correlate with a high anthocyanin content, which is coherent with the TAC of the samples. On the other hand, Sample 1 (north of Montevideo) correlated with the myricetin glycosides (C6, C8 and C9), while sample 3 had a stronger correlation to the quercetin derivatives. This PCA diagram explains 88.11% of the total variance (PC1 = 68.58% and PC2 = 19.53%).

**Figure 3:**
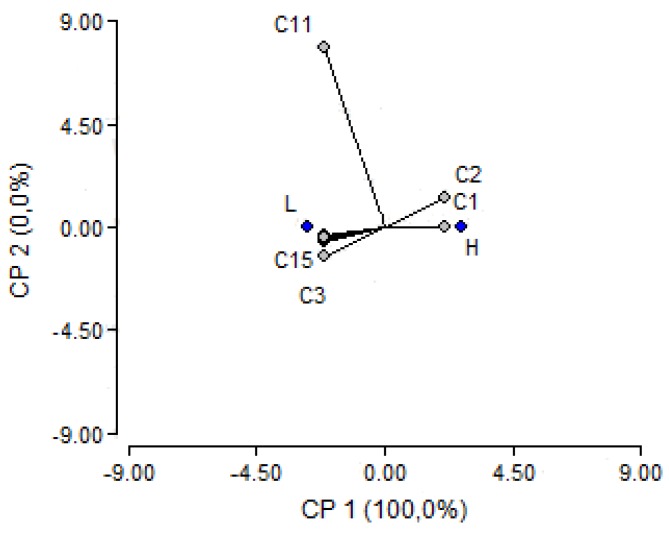
PCA where all identified compounds are classified according to low (L) and high (H) ORAC results.

Samples 1, 4, 5, 6 and 7 were labeled as high ORAC samples (H), while samples 2 and 3 were labeled as low ORAC samples (L). The compounds that classified the H samples were the anthocyanins while the flavonols classified the L samples, see Figure 3.

In order to predict the ORAC and DPPH values of a new sample based on the concentration of the identified compounds, we performed a stepwise multivariate linear regression analysis, the equations described below were obtained:

ORAC = 69825.66 − 552.22[C11] + 378.91[C5] (*R*^2^ = 0.7345, *p*-value = 1.876 × 10^−5^, RSE = 9050 on 15 Degrees of Freedom) (1)

DPPH = 14108.99 − 97.55[C11] + 168.64[C9] − 246.19[C15] + 421.48[C14] − 260.30[C5] − 367.10[C8] − 80.43[C10] + 287.09[C4] − 527.05[C3] − 80.35[C6] (*R*^2^ = 0.9995, *p*-value = 8.487 × 10^−12^, RSE = 238.3 on 7 Degrees of Freedom) (2)

An increased number of reports have shown the non-additivity of the antioxidant properties of polyphenols, in the ORAC and DPPH assays. Particularly, they can act negatively on the overall value of the measurement, depending on the certain mixture of polyphenols and the point they enter in the reaction chain cycle, either as oxidants or reductants. The anthocyanins, due to their positive formal charge, are kinetically more reactive than the neutral polyphenols when reacting with DPPH. The reaction chain thus triggered could foster negative contributions of the quercetin derivatives, but positive ones from the myricetin ones, based on the positive or negative contributions shown in Equation (2). These mixture effects were “partly explained by regeneration mechanisms between antioxidants, depending on the chemical structure of molecules and on the possible formation of stable intermolecular complexes” [31]. Further work is required to understand the global behavior of complex mixtures of different classes of polyphenols in these widely used bench-top bioassays.

## 4. Conclusions

The samples from different Uruguayan locations were very similar on their composition but presented different levels of antioxidant activity. The sample collected in 2014 in the north of Montevideo showed the highest ORAC and DPPH values. Several samples were collected until April 2016 and the evaluated. There is no clear relationship between the TAC and TFC values and the bioactivity studied; due to the presence of secondary metabolites with antioxidant activity in Pitanga fruit, for example carotenoids [6]. However, we could identify the phenolic compounds responsible for the separation between samples using PCA diagrams. Furthermore, the different location samples were characterized by specific types of phenolic compounds.

The sample collected in April 2016 (sample 7) showed a TPC three times higher than the sample collected in December 2015 (sample 6) probably because of the ripening during summer season where light exposure enhances the quality of the fruits.

In contrast to the results obtained by Celli et al. [5], the main compounds identified amongst all flavonols were myricetin derivatives and not quercetin derivatives, these results can be used as a characteristic of Uruguayan Pitanga fruits, with high antioxidant capacity.

## Figures and Tables

**Table 1:** Water content calculated by weighing the samples before and after lyophilization.

Sample	Location/Sampling Date	% Water (% *w*/*w*) ^1^
1	North of Montevideo/December 2014	68.1 ^c,d^ ± 0.9
2	South of Montevideo/December 2014	63.6 ^b,c^ ± 1.7
3	Paysandú/December 2014	66.1 ^b,c,d^ ± 0.9
4	Canelones/December 2014	46.4 ^a^ ± 6.4
5	North of Montevideo/November 2015	66.5 ^b,c,d^ ± 4.1
6	North of Montevideo/December 2015	58.3 ^b^ ± 0.6
7	North of Montevideo/April 2016	73.0 ^d^ ± 2.7

^1^ Means (*n* = 3) followed by different letters are significantly different at *p* < 0.05 according to Tukey’s test.

**Table 2:** Phenolic compounds identified and quantified (µmol/100 g dry weight) in different samples ^1^.

Compound ^2^	Rt	[M − H]^+^	MS^n^	Sample 1	Sample 2	Sample 3	Sample 4	Sample 5	Sample 6	Sample 7
**C1**	29.8	465	303	19.99 ^a^ ± 3.34	373.24 ^c^ ± 3.98	ND ^3,a^	381.38 ^c^ ± 31.81	282.99 ^b^ ± 24.49	215.53 ^b^ ± 19.42	485.81 ^d^ ± 26.25
**C2**	33.8	449	287	115.01 ^a^ ± 8.56	1757.29 ^d^ ± 18.34	150.45 ^a^ ± 0.25	987.77 ^c^ ± 49.63	916.35 ^b,c^ ± 85.15	706.78 ^b^ ± 68.87	2248.68 ^e^ ± 98.76
		**[M − H]^−^**								
**C3**	48.4	631	479, 317, 271	8.07 ^d^ ± 1.15	14.18 ^f^ ± 0.34	10.67 ^e^ ± 0.34	ND ^3,a^	3.63 ^b,c^ ± 0.08	2.41 ^b^ ± 0.57	4.94 ^c^ ± 0.36
**C4**	53.6	479	317, 271	34.33 ^c^ ± 1.28	46.41 ^d^ ± 0.43	56.53 ^e^ ± 1.11	6.11 ^a^ ± 0.18	12.97 ^b^ ± 1.52	8.77 ^a^ ± 0.86	17.05 ^b^ ± 2.14
**C5**	54.7	479	317, 271	32.37 ^d^ ± 2.52	35.82 ^d^ ± 0.34	22.59 ^c^ ± 1.49	6.53 ^a,b^ ± 0.50	4.85 ^a^ ± 1.76	7.57 ^a,b^ ± 0.78	10.25 ^b^ ± 2.45
**C6**	56.8	449	317, 271, 179	23.44 ^d^ ± 0.26	6.79 ^b,c^ ± 0.51	9.39 ^c^ ± 4.91	1.22 ^a^ ± 1.46	2.15 ^a,b^ ± 0.24	1.52 ^a^ ± 0.18	3.33 ^a,b^ ± 0.35
**C7**	58.0	615	463, 301, 271	8.45 ^c^ ± 0.09	11.10 ^d^ ± 0.64	16.87 ^e^ ± 0.43	0.92 ^a^ ± 0.81	7.61 ^b,c^ ± 0.65	5.27 ^b^ ± 0.75	11.59 ^d^ ± 1.19
**C8**	61.0	449	317, 271, 179	49.91 ^d^ ± 3.46	4.57 ^a,b^ ± 0.94	26.30 ^c^ ± 0.85	2.09 ^a^ ± 0.20	5.33 ^a,b^ ± 1.10	3.96 ^a^ ± 0.78	8.34 ^b^ ± 1.11
**C9**	61.9	463	317, 271, 179	378.69 ^f^ ± 10.72	138.43 ^d^ ± 3.37	243.25 ^e^ ± 1.20	16.81 ^a^ ± 1.04	41.63 ^b^ ± 3.49	29.16 ^a,b^ ± 3.76	56.62 ^c^ ± 3.33
**C10**	62.9	463	301	29.25 ^c^ ± 2.13	64.65 ^d^ ± 2.39	167.25 ^e^ ± 0.00	4.11 ^a^ ± 1.50	14.97 ^b^ ± 0.59	8.17 ^a^ ± 0.66	18.48 ^b^ ± 1.05
**C11**	63.5	463	301	38.30 ^b^ ± 2.65	71.26 ^c^ ± 4.27	101.54 ^d^ ± 3.59	13.11 ^a^ ± 3.81	8.22 ^a^ ± 0.80	8.32 ^a^ ± 1.02	10.61 ^a^ ± 0.20
**C12**	64.5	433	301	8.97 ^c^ ± 1.24	10.59 ^c^ ± 0.04	19.64 ^d^ ± 0.30	2.57 ^a,b^ ± 0.14	2.41 ^a,b^ ± 0.48	1.75 ^a^ ± 0.42	4.17 ^b^ ± 1.35
**C13**	64.9	433	301	28.44 ^d^ ± 1.75	8.71 ^c^ ± 2.43	55.81 ^e^ ± 0.38	6.55 ^a^ ± 1.35	11.36 ^a,b^ ± 2.30	6.79 ^a^ ± 0.96	16.48 ^b,c^ ± 1.55
**C14**	65.4	433	301	36.12 ^d^ ± 0.43	36.21 ^d^ ± 0.13	92.95 ^e^ ± 0.17	3.37 ^a^ ± 0.51	15.12 ^b^ ± 2.32	9.98 ^b^ ± 2.93	22.92 ^c^ ± 2.90
**C15**	65.7	447	301	80.74 ^d^ ± 0.13	93.13 ^e^ ± 1.54	224.17 ^f^ ± 0.04	15.21 ^a^ ± 0.33	34.62 ^b^ ± 3.82	26.44 ^b^ ± 4.27	53.22 ^c^ ± 3.34

^1^ Means (*n* = 3) in the same rows followed by different letters are significantly different at *p* < 0.05 according to Tukey’s test. ^2^ Compounds: anthocyanins quantified at 520 nm: **C1**: delphinidin-3-hexoside, **C2**: cyanidin-3-hexoside. Flavonols quantified at 360 nm: **C3**: myricetin-galloyl-hexoside, **C4**: myricetin-hexoside 1, **C5**: myricetin-hexoside 2, **C6**: myricetin-pentoside 1, **C7**: quercetin galloyl hexoside, **C8**: myricetin-pentoside 2, **C9**: myricetin-rhamnoside, **C10**: quercetin hexoside 1, **C11**: quercetin hexoside 2, **C12**: quercetin pentoside 1, **C13**: quercetin pentoside 2, **C14**: quercetin pentoside 3 and **C15**: quercetin rhamnoside. ^3^ ND = not detected.

**Table 3:** Total anthocyanin content (TAC), total quercetin derivatives content (TQC), total myricetin derivatives content (TMC), total flavonol content (TFC), total catechol derivatives content (TCC), total pyrogallol derivatives content (TPC) and total polyphenol content (TPPC) results expressed as µmol/100 g dry weight.

	Sample 1	Sample 2	Sample 3	Sample 4	Sample 5	Sample 6	Sample 7
TAC	134.98 ^a^ ± 11.90	2130.53 ^d^ ± 14.35	150.45 ^a^ ± 0.24	1369.15 ^c^ ± 79.26	1199.35 ^b,c^ ± 109.47	922.32 ^b^ ± 87.78	2734.50 ^e^ ± 125.00
TQC	230.26 ^d^ ± 1.61	308.45 ^e^ ± 8.03	678.25 ^f^ ± 4.14	45.84 ^a^ ± 2.98	94.33 ^b^ ± 8.56	66.70 ^a^ ± 7.42	137.44 ^c^ ± 11.21
TMC	526.83 ^f^ ± 17.08	246.20 ^d^ ± 3.43	368.79 ^e^ ± 6.24	32.76 ^a^ ± 1.65	70.54 ^b^ ± 8.14	53.39 ^a,b^ ± 6.56	100.50 ^c^ ± 9.57
TFC	757.09 ^f^ ± 18.69	554.65 ^e^ ± 11.47	1047.04 ^g^ ± 10.38	78.60 ^a^ ± 2.50	164.87 ^c^ ± 14.57	120.08 ^b^ ± 7.39	237.94 ^d^ ± 17.48
TCC	345.25 ^a^ ± 10.18	2065.75 ^d^ ± 10.30	828.70 ^b,c^ ± 4.38	1033.62 ^c^ ± 49.24	1010.68 ^c^ ± 92.65	773.48 ^b^ ± 65.14	2386.12 ^e^ ± 100.13
TPC	546.82 ^c^ ± 20.42	619.43 ^c^ ± 7.42	368.79 ^b^ ± 6.28	414.13 ^b^ ± 31.28	353.54 ^a,b^ ± 32.23	268.92 ^a^ ± 25.93	586.32 ^c^ ± 35.30
TPPC	892.07 ^a^ ± 30.59	2685.18 ^d^ ± 2.89	1197.49 ^b,c^ ± 10.62	1447.75 ^c^ ± 77.27	1364.21 ^c^ ± 124.02	1042.40 ^a,b^ ± 90.94	2972.43 ^d^ ± 135.41

Means (*n* = 3) in the same rows followed by different letters are significantly different at *p* < 0.05 according to Tukey’s test.

**Table 4:** Oxygen Radical Absorbance Capacity (ORAC) and 2,2-diphenyl-1-picrylhidracyl (DPPH) antioxidant activity results for each sample.

Assay	Sample 1	Sample 2	Sample 3	Sample 4	Sample 5	Sample 6	Sample 7
ORAC	57,440 ^b,c^ ± 1090	44,510 ^a,b^ ± 460	22,800 ^a^ ± 300	57,560 ^b,c^ ± 10,040	57,400 ^b,c^ ± 16,380	82,340 ^c^ ± 9070	72,550 ^c^ ± 15,280
DPPH	44,170 ^b^ ± 4480	11,940 ^a^ ± 2200	10,070 ^a^ ± 1200	12,200 ^a^ ± 650	15,430 ^a^ ± 70	12,960 ^a^ ± 1250	13,950 ^a^ ± 560

Means (*n* = 3) in the same rows followed by different letters are significantly different at *p* < 0.05 according to Tukey’s test.
